# Obstructive sleep apnea and hypertension; critical overview

**DOI:** 10.1186/s40885-024-00276-7

**Published:** 2024-08-01

**Authors:** Younghoon Kwon, William S Tzeng, Jiwon Seo, Jeongok Gang Logan, Marijana Tadic, Gen-Min Lin, Miguel Angel Martinez-Garcia, Martino Pengo, Xiaoyue Liu, Yeilim Cho, Luciano F. Drager, William Healy, Geu-Ru Hong

**Affiliations:** 1 Department of Medicine, University of Washington, Seattle, WA USA; 2 Division of Cardiology, Severance Cardiovascular Hospital, Yonsei University College of Medicine, 50–1 Yonsei-Ro, Seodaemun-Gu, Seoul, 03722 Republic of Korea; 3 Department of Acute & Specialty Care, University of Virginia School of Nursing, Charlottesville, VA USA; 4 Klinik Für Innere Medizin II, Universitätsklinikum Ulm, Ulm, Germany; 5 Department of Medicine, Hualien Armed Forces General Hospital, Hualien, Taiwan; 6 Department of Medicine, Tri-Service General Hospital, National Defense Medical Center, Taipei, Taiwan; 7 Department of Pneumology, Polytechnic and University La Fe Hospital, Valencia, Spain; 8 Department of Medicine and Surgery, University of Milano-Bicocca, Milan, Italy; 9 Department of Cardiology, Istituto Auxologico Italiano IRCCS, S.Luca Hospital, Milan, Italy; 10 Johns Hopkins School of Nursing, Johns Hopkins University, Baltimore, MD USA; 11 Department of Sleep Medicine, University of Washington, Seattle, WA USA; 12 Department of Sleep Medicine, Veteran’s Affairs Puget Sound Healthcare System, Seattle, WA USA; 13 Hypertension Unit, Heart Institute, University of Sao Paulo Medical School, Sao Paulo, Brazil; 14 Division of Pulmonary, Critical Care, Sleep Medicine, Medical College of Georgia, Augusta, GA USA; 15 Division of Cardiology, University of Washington, Seattle, WA USA

**Keywords:** Obstructive sleep apnea, Continuous positive airway pressure, Hypertension, Blood pressure

## Abstract

Obstructive sleep apnea (OSA) and hypertension are two important modifiable risk factors for cardiovascular disease and mortality. Numerous studies have highlighted the interplay between these two conditions. We provide a critical review of the current literature on the role of the OSA as a risk factor for hypertension and its effect on blood pressure (BP). We discuss several key topics: the effect of OSA on nocturnal BP, BP response to continuous positive airway pressure (CPAP) treatment, CPAP effect on BP in refractory hypertension, the role of OSA in BP variability (BPV), and maladaptive cardiac remodeling mediated by OSA’s effect on BP. Finally, we discuss the unique aspects of ethnicity and social determinants of health on OSA with a focus on Asian populations and the disparity in BP control and cardiovascular outcomes.

## Background

Hypertension is one of the most important risk factors for cardiovascular disease and mortality. Numerous studies have suggested the crucial role of sleep in blood pressure (BP) regulation. Recently, sleep has also been included by the American Heart Association as one of the 8 elements essential for cardiovascular health [1]. Obstructive sleep apnea (OSA) is a common sleep disorder characterized by repetitive upper airway obstruction during sleep and is a well-established cause of poor sleep. The goal of this paper is to provide a critical overview of the interplay between OSA and hypertension by summarizing the existing evidence regarding pathophysiological link and implications of OSA treatment with a special focus on the areas that have not been sufficiently discussed in the literature. We further discuss gaps and future directions on this topic.

### OSA and hypertension: epidemiology

Hypertension is one of the most well-recognized modifiable risk factors for cardiovascular morbidity and mortality [2]. Although the etiology of hypertension is largely idiopathic (“essential”), genetic predisposition, lifestyle factors including diet, exercise, smoking, and stress, and comorbidities such as metabolic dysfunction and renal dysfunction have an important pathophysiological link to hypertension [3, 4].

Sleep is another important lifestyle factor that is increasingly recognized to have an impact on BP regulation [5]. In particular, numerous studies have suggested that a pathological sleep disorder such as OSA is an independent risk factor for hypertension. OSA is a common sleep disorder affecting nearly 10–15% of women and 15–30% of men [5, 6]. It is a spectrum of conditions that includes a wide range of severity of upper airway obstructions during sleep. OSA impairs sleep quality due to frequent episodes of OSA-related arousal and compromises sleep architecture. Patients with OSA exhibit a high prevalence of hypertension and those with hypertension have a high prevalence of OSA. Furthermore, several prospective studies hint that OSA may increase the risk of incident hypertension. A landmark study from the Wisconsin Sleep Cohort study (WSCS) showed that OSA is associated with an increased future risk of hypertension independent of age, sex, body mass index (BMI), and other potential confounders at baseline in a dose-dependent manner; the more severe the OSA, the higher risk of incident hypertension [7]. On the contrary, in some studies the association between OSA and incident hypertension attenuates and becomes insignificant after adjusting for potential confounders such as age, sex, and BMI. For example, the association between OSA and incident hypertension in the Sleep Heart Health Study (SHHS) was no longer significant after controlling for BMI when followed over 5 years [8]. This discrepancy can be explained in part by the age difference between the cohorts in either study; SHHS patients were much older than those in WSCS (60 years versus 47 years) [9]. The strength of the association between OSA severity and incident hypertension appears to decline with age. In SHHS, a reduced correlation between OSA severity and incident hypertension above an age cut-off of 60 years was demonstrated. Similarly, in the Vitoria Sleep Cohort (VSC), there was a dose-dependent association between OSA severity and incident hypertension in 1,180 middle- and old-aged patients followed for 7.5 years, but the association was reduced and no longer significant after controlling for age [10]. Other reasons for the disparate results between WSCS, SHHS, and VSC may be due to population differences and differences in assessments for OSA. For example, the reference in WSCS used an AHI of 0/hr, whereas in SHHS, the reference used a range of AHI: 0–4.9/hr. In contrast, the reference in VSC used 0–2.9/hr (1st quartile for Respiratory Disturbance Index (RDI)).

In Asian adults, daytime BP was associated with age and OSA severity in a Taiwanese cross-sectional study [11]. In this study, the BP and age were correlated in patients with mild to moderate OSA, but not in those with severe OSA. When compared to the Westerners, Asians were found to have a greater prevalence of increased nighttime BP or morning BP surge, as assessed by ambulatory BP monitoring (ABPM). In a Japanese study on 38 adult patients with severe OSA, both daytime and nighttime BPs were significantly reduced and 15 of 22 non-dippers became dippers after continuous positive airway pressure (CPAP) treatment for 3 days [12]. However, a prospective longitudinal study on the association between OSA and incident hypertension in Asian populations is still lacking.

### OSA and hypertension: pathophysiology

The pathophysiology of hypertension in OSA is complex and involves both direct and indirect mechanisms. The acute impact of OSA is well established. Each OSA event leads to varying degrees of cascades of cardiovascular and central nervous system responses including sympathetic surge, hypoxemia, hypercapnia, intrathoracic pressure swings and arousal [13–15]. Sympathetic responses can be manifested as an abrupt BP surge and peripheral vasoconstriction [16]. The mechanism of sympathetic surge is unclear but may be related to hypoxia-induced stimulation of the carotid body chemoreceptors, causing reflex sympathetic stimulation of the medullary cardiorespiratory centers [17]. Moreover, the intrathoracic pressure swing generated when massive efforts to breath against an obstructed airway are made can elicit sympathetic surge [18]. Intriguingly, increased sympathetic activity has been observed in patients with OSA during daytime [19, 20].

**Fig. 1:**
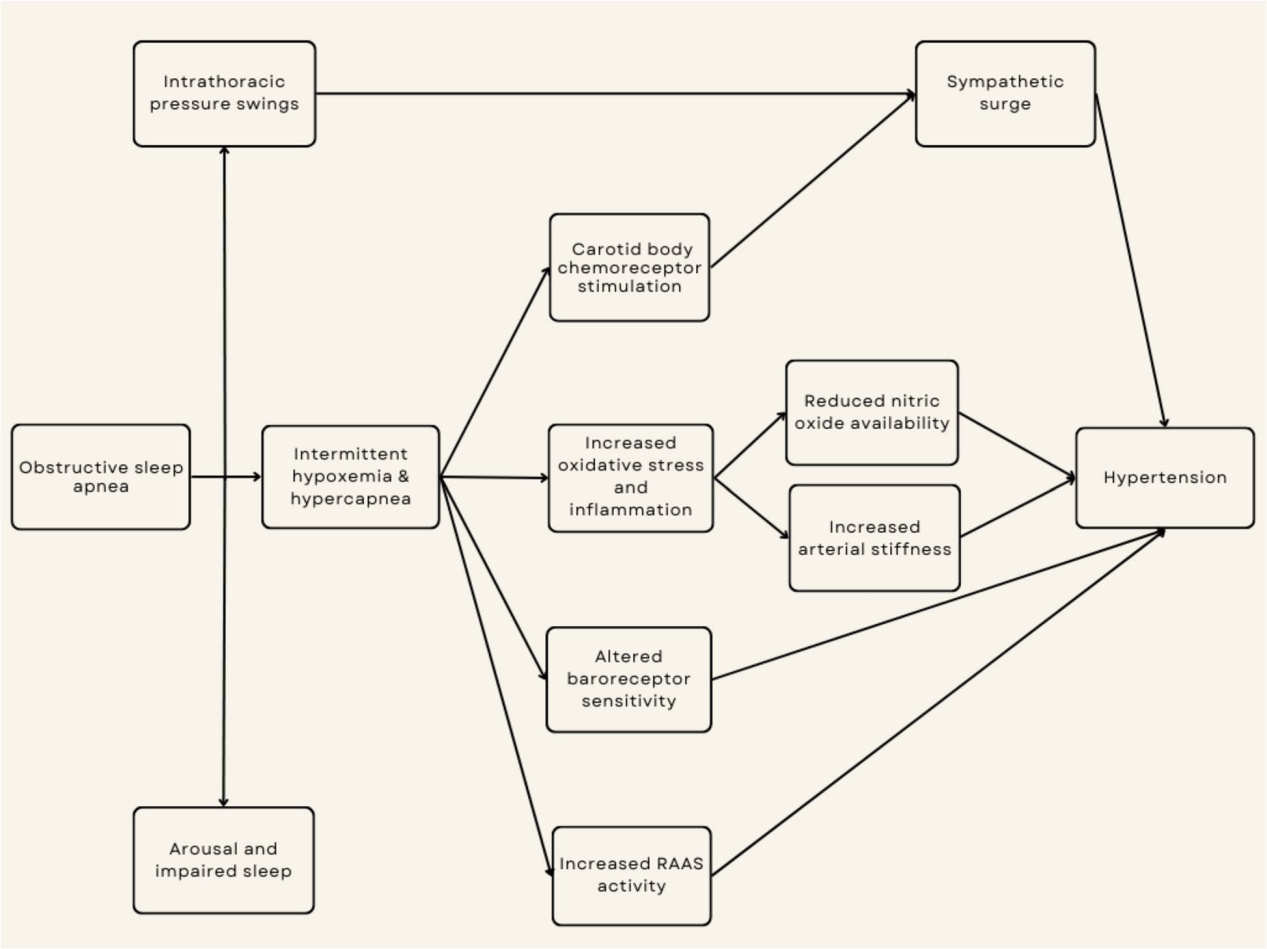
Mechanisms that may be responsible for hypertension in obstructive sleep apnea: Obstructive sleep apnea increased arterial stiffness, increased renin–angiotensin–aldosterone activity, and altered baroreceptor reflexes

In the long term, OSA is associated with reduced endothelial nitric oxide availability, whereas oxidative stress and inflammation are enhanced [13–15, 21]. Other mechanisms that may be responsible for hypertension in OSA are increased arterial stiffness, increased renin–angiotensin–aldosterone activity, and altered baroreceptor reflexes (Fig. 1). Not only does OSA mediate hypertension through these pathways, but it may also regulate BP through these same mechanisms [22, 23]. These effects in combination with other common risk factors that accompany patients with OSA such as obesity and metabolic dysfunction can exert negative effects on BP regulation.

### OSA and BP variability

BP is a biological signal characterized by marked fluctuations occurring within a 24-h period. This BP variability (BPV) is regulated by several mechanisms that balance adequate organ perfusion with possible damage from excessively high BP [24]. OSA may disrupt these regular fluctuations through its complex pathophysiologic relationship with hypertension as previously highlighted; excessive BPV over time may reflect an alteration in regulatory mechanisms such as poor autonomic control or vascular damage [25]. Furthermore, more studies are supporting sustained BPV as a strong and independent risk factor for cardiovascular disease and target organ damage beyond mean BP [26–28].

BPV is studied in the very short-term (beat-to-beat), short-term (within 24 h), mid-term (day-to-day), and long-term (week-to-week or month-to-month, also called visit-to-visit BPV) [22, 29]. Beat-to-beat BP measurements have been commonly used to measure cardiac baroreflex sensitivity [30]. Previous studies have demonstrated that beat-to-beat BPV is higher in OSA compared with non-OSA subjects [25, 31] and have suggested that this may reflect poor baroreflex control in patients with OSA [25]. Beat-to-beat BPV in patients with OSA may also provide unique information on BP behavior in relation to sleep apneic events. Multiple factors including patient-level underlying factors and sleep apnea event characteristics are expected to determine the severity of BP surge following sleep apneic events. However, some studies have shown a stronger relationship between apnea length [32] and degree of desaturation with the highest BPs typically observed during REM sleep [16, 33, 34]. Short-term BPV has been evaluated mainly as changes in mean BP from day to night in the form of dipping/non-dipping or morning BP surge. Yet, a few studies have shown that nocturnal systolic BPV and 24-h systolic BPV are also significantly increased in hypertensive OSA patients when compared with hypertensive non-OSA patients [35–37]. As for mid-term and long-term BPV, limited studies are available. Studies have thus far demonstrated that patients with OSA had increased visit-to-visit BPV and that patients with good adherence to CPAP therapy had the significantly reduced visit-to-visit BPV [38]. Further evidence is needed to better characterize the impact of OSA on BP regulation, particularly the mid and long-term. Assessment and treatment of BPV in OSA patients may add to the risk stratification and the evaluation of CPAP treatment [39].

### OSA and nocturnal dipping

OSA may also contribute to impaired normal BP dipping. Previous studies have shown that a non-dipping pattern is found in 48% to 84% of patients with OSA. Its frequency and related nighttime BP also increases with OSA severity [40–42]. Non-dipping BP patterns also have utility in predicting the presence of OSA, despite their nonspecific nature. Crinion et al. found that moderate to severe OSA was associated with a 5.5-fold increased chance of having non-dipping BP after adjusting for potential confounders in patients with hypertension. [43] Interestingly, Genta-Pereira et al. found that the type of BP dipping may modify the prediction of having OSA. [44] In this study, reverse systolic BP dippers were independently associated with OSA, whereas both reduced and reverse diastolic dippers increased the likelihood of OSA. Surprisingly, the presence of snoring and two largely used sleep questionnaires only modestly improved the accuracy for predicting OSA [44].

### Evaluating BP in OSA through ambulatory BP monitoring

The close association between OSA and hypertension highlights how BP assessment is crucial in patients with OSA whether they have an established diagnosis of hypertension or not. Capturing the myriad hemodynamic changes promoted by respiratory events, however, is not practical even in a sophisticated clinical sleep laboratory. 24-h ABPM in OSA can provide important insights and help identify patients with masked hypertension, isolated nocturnal hypertension, and daytime hypertension with pronounced nocturnal hypertension [29]. It has been observed that patients with newly diagnosed OSA have significantly higher 24-h systolic BPV when compared to those without OSA [35]. Such increased BPV may cause office BP to be less reliable, highlighting the benefit of the ABPM in patients with OSA [45].

Continuous beat to beat BP monitoring, though not readily available, also provides more insights into the pathophysiology of the OSA in relation to nocturnal hypertension. More specifically, if it is coupled with sleep studies, OSA related BP surge information can be derived [46]. This in turn, will help us understand the severity of OSA and better predict cardiovascular outcomes.

### Impact of OSA treatment on BP

Over the last three decades, several research groups worldwide have tried to investigate the effect of OSA treatment on BP with conflicting results. While many studies have confirmed a modest but significant BP reduction, others have failed to show BP improvements in patients treated either with CPAP or mandibular advancement device [47]. In general, the impact of OSA treatment on in-office BP is modest – around 2 to 3 mmHg – and most studies focus on the effects of CPAP [48, 49]. This reduction is more pronounced for nocturnal BP [49–51]. However, these same meta-analyses also highlight important heterogeneity between studies. These differences were confirmed when a risk of bias analysis was performed, making it difficult to compare the results of the clinical trials. This suggests that there are subgroups or phenotypes of OSA patients who exhibit a more robust BP reduction when treated for OSA. For example, Castro Gratinoni et al. showed that the presence of a non-dipping pattern in patients with OSA was a predictor of better BP reduction (Nighttime mean BP change: non-dipper/low heart rate =  − 6.2 ± 8.32 mmHg vs. dipper/low heart rate = 6 ± 6.97 mmHg) in response to CPAP. [45] Another intriguing finding that is still poorly understood is that there was a significant correlation between the degree of daytime sleepiness and the reduction in daytime SBP/DBP in patients with OSA [52]. Table 1 depicts the current characteristics that predict a higher impact on reducing BP after OSA treatment. These predictors need to be validated in longitudinal studies to determine if BP improvements in specific subgroups can be translated into a reduction of cardiovascular events.

**Table 1:** Predictors of good blood pressure response to OSA treatment

Patients with good adherence to OSA treatment
Patients with excessive daytime sleepiness
Patients with resistant hypertension
Patients with refractory hypertension
Patients with non-dipping BP profile (mainly when higher heart rate is present)

Some additional insights could come from the ANDANTE project [53] (a Worldwide Individual Data Meta-Analysis of the Effect of Sleep Apnea Treatment on Blood Pressure) and the MORPHEOS trial [54] (a multicenter randomized controlled trial designed to evaluate the BP lowering effects of CPAP in OSA patients with uncontrolled hypertension under pill counting). Future studies will need to consider the pitfalls and limitations of previous studies such as suboptimal CPAP compliance.

Additional subgroups that may respond well to CPAP treatment include resistant hypertension (RH) and refractory hypertension (RFH). RH is defined as BP that remains uncontrolled despite the use of at least three antihypertensive drugs or BP that requires at least 4 antihypertensive drugs to control [55]. RFH is defined as BP that remains uncontrolled despite the use of at least 5 antihypertensive drugs [56]. The prevalence of RH and RFH in patients with OSA is greater than 70% and 90% respectively [57, 58]. A recent meta-analysis on the effect of CPAP in RH concluded that good CPAP adherence significantly reduces both 24-h systolic and diastolic BP values by 4–5 mmHg, especially at night [59]. Other randomized studies showed up to 10 mmHg of the BP reduction [60–62]. These decreases are seen more prominently (8–10 mmHg) in patients with RFH [63]. Of course, CPAP adherence was a crucial factor associated with more significant reductions in nocturnal BP [63]. Therefore, patients with RH not explained by other causes should be referred for sleep study to rule out OSA.

#### OSA, hypertension, and its impact on cardiac remodeling

Hypertension is an important contributor to cardiac remodeling. OSA’s adverse cardiovascular effect is thought to be mainly mediated through hypertension although a direct effect on the myocardium likely exists. Studies have shown that OSA is associated with left ventricular (LV) hypertrophy, LV diastolic dysfunction, and impairment in LV mechanics [64–66]. The first studies on this topic using conventional echocardiography reported baseline OSA was associated with reduced LV systolic function by LV ejection fraction (EF) by echocardiography obtained over an average of 18-year follow up [64]. Since then, a prospective randomized sham-controlled trial showed that treatment with CPAP for 3 months in patients with severe OSA resulted in significantly improved LV diastolic function compared to sham treatment [67]. The development of new imaging techniques such as speckle tracking and 3D echocardiography provided more insightful information on LV and right ventricle (RV) systolic function in OSA patients [67–70]. It also provided information on the efficacy of treatment of OSA with CPAP on reverse cardiac remodeling [70]. These studies suggested that LV mechanics, particularly global longitudinal strain (GLS), might be the most sensitive marker of initial subtle changes and LV remodeling in OSA patients. They also suggest that there are beneficial LV changes after starting CPAP therapy. However, separating out the effect of common comorbidities – hypertension, obesity, and diabetes – on cardiac remodeling is difficult. Most studies tried to adjust the resultant cardiac changes in parameters such as LV hypertrophy, but it is difficult to avoid the influence of these comorbidities as confounding factors. A recent meta-analysis showed that LV GLS is not only significantly decreased in OSA patients, but there appears to be a dose–response with OSA severity even among normotensive OSA patients [71]. Another meta-analysis that investigated the influence of CPAP reported that CPAP treatment was related to positive effects on LV and RV function in patients with OSA, particularly in terms of LV and RV GLS [72]. These results suggest that the assessment of cardiac mechanics by speckle tracking echocardiography should be included in the routine echocardiographic examination of patients with OSA.

There are several possible mechanisms that could clarify the relationship between OSA and cardiac remodeling. The overactivation of the sympathetic nervous system during repeated episodes of apnea results in episodic BP surge, regardless of the presence of daytime hypertension. Such nocturnal hypertension is a potent mediator of cardiac remodeling in both the LV and RV. Moreover, elevated BP associated with OSA may act as negative feedback, resulting in worsened OSA, which may accelerate LV and RV remodeling. Intermittent hypoxia stimulates an inflammatory reaction involving proinflammatory cytokines, growth factors, and oxidative stress. The activation of both humoral systems induces proliferation of extracellular matrix, interstitial myocardial fibrosis, and an increase in myocardial stiffness, which contributes to the development of LV and RV diastolic dysfunction and impairment of GLS. Interventricular dependence could be an additional mechanism responsible for LV and RV changes due to increased pulmonary arterial pressure that retrogradely transfers from the RV through the pulmonary circulation to the LA causing increased LV filling pressure and LV diastolic dysfunction. Cardiac remodeling can be clinically manifested as heart failure or arrythmia. Several studies suggested that OSA is an independent risk factor for heart failure and atrial fibrillation in patients without other underlying cardiac disorders [73–75].

#### OSA, arterial stiffness, and LV hemodynamics

Previous evidence has shown that sympathetic activation, systemic inflammation, and endothelial dysfunction are significantly increased in patients with OSA compared to those without OSA. These adverse consequences are closely related to BP, ventricular hemodynamics, and arterial stiffness. Moreover, elevated BP, hemodynamic changes, and arterial stiffness also mutually affect each other. Arterial stiffening causes the early return of reflected waves to elevate systolic BP and pulse pressure. Further increases in BP promote matrix synthesis, causing subsequent increases in vascular thickness and stiffening and increasing the load of stiff components within the arterial wall [76]. Ventricular-vascular uncoupling is also associated with increased systolic BP and subclinical LV remodeling [77, 78].

Recent studies have shown that CPAP treatment significantly improves stroke volume, ventricular-vascular coupling, and arterial stiffness measured by pulse wave doppler in patients with OSA [67]. Although the exact mechanism of these improvements is still unclear, some evidence suggests that CPAP therapy improves endothelial dysfunction, decreases oxidative stress markers, and restores ventricular hemodynamics and sympathetic nervous system activity [79]. OSA promotes the clinical combination of hypertension, ventricular-vascular uncoupling, and arterial stiffness, which is a major step toward the development of cardiovascular disease. Therefore, measuring the LV hemodynamics and arterial stiffness, as well as BP monitoring, can be an important clinical tool for predicting cardiovascular complications and treatment efficacy in patients with OSA.

### OSA-hypertension and the role of social and racial-ethnicity

A burgeoning body of literature suggests that the relationship between OSA and hypertension is substantially affected by social determinants of health [80, 81]. Racial difference is a significant factor contributing to the disparities in BP outcomes among adults with OSA. One such study suggests that untreated moderate or severe OSA may be related to a two-fold higher odds of resistant hypertension in Black adults [82]. In a nationally representative sample, probable OSA was associated with an over fourfold increased odds of predicting hypertension among overweight Black participants (95% confidence interval [CI], 1.86–12.03) and a twofold increased odds among obese Hispanic participants (95% CI, 1.16–3.49) [81]. Although Asians have comparable rates of OSA compared to Whites, Asians have a higher prevalence of increased nighttime BP or morning BP surge [83]. This implies that Asians may be more susceptible to poor BP outcomes in the setting of OSA than their white counterparts [83, 84]. Indeed, studies have observed a link between OSA and abnormal dipping patterns of BP in Asian adults [83]. However, these findings were primarily established based on Japanese populations [12, 85] and more studies are needed to examine the relationship between OSA and hypertension in other Asian sub-groups given the substantial heterogeneity across Asian backgrounds. Other socioeconomic factors that increase the burden of OSA and hypertension may include living in a disadvantaged neighborhood [86, 87], lacking health insurance [88], and not being married [89, 90].

### Addressing health disparities in OSA to improve hypertension care

Considering the high prevalence of OSA and hypertension, as well as the health consequences associated with these two conditions, future investigations should focus on identifying the social determinants that underlie the OSA-hypertension relationship. These important inquiries may assist with developing tailored interventions to reduce the health disparities among high-risk populations such as racial and ethnic minority groups. Improving identification and treatment of OSA in socioeconomically disadvantaged ethnicities will require novel and innovative care delivery models. One method may be simplifying the screening process. With technological advances, OSA evaluation can be typically performed with a home sleep apnea test. Telemonitoring can be an effective tool to follow up on patients requiring therapy. Integration of sleep services into primary care or other specialties may increase access for these populations. For example, incorporating OSA screening into cardiology practice may simplify the referral and treatment process.

In the US, the American Academy of Sleep Medicine has signaled national interest in this area through development of the Specialty Practice Accreditation program for cardiology clinics. This simplified referral and therapy approach may benefit blacks, who are most vulnerable to hypertension and its untoward complications [91]. However, whether such efforts will improve OSA care in the socioeconomically disadvantaged population as a whole remains to be seen. Not only must changes be implemented at the site of practice, but there must also be reform at the level of manufacture and distribution of the treatment modalities such as CPAP equipment to make therapy more readily accessible. Efforts to continue to streamline and simplify the diagnosis, treatment, and reimbursement for CPAP equipment will improve outcomes in vulnerable populations most susceptible to social and economic hardships who also have the most disparity in cardiovascular outcomes.

### Gaps and future prospects

While prospective longitudinal studies have linked OSA to increased risk of hypertension [7, 92], such a finding has not been consistent [8, 10]. In patients with OSA, OSA treatment with CPAP has been shown to improve BP but the effect has been modest. Symptomatic phenotype may play a role in determining the benefit of CPAP on BP wherein the BP reduction is less pronounced in non-sleepy OSA patients [93–97]. The impact of OSA on nocturnal BP requires more in-depth investigation. This is because of the inherent limitation of using conventional ABPM device, which only yields intermittent BP readings in sleep. BP response to OSA itself may be an important indicator for adverse cardiovascular prognosis. Beat to beat BP measurement or hypoxia triggered BP monitoring will provide deeper insights into this [98]. This can be possible through direct BP measurement [16], estimation using other available physiological measurements such as pulse arrival time [99, 100], or potentially photoplethysmography signals derived from wearable devices [101–103]. More studies are essential to examine the clinical value of continuous BP monitoring in sleep.

There is a shift from a single in-lab polysomnography to multi-night portable home sleep studies and beyond (wearables). Such a transition will allow us to examine the role of sleep on BP control at a much higher dimension. Finally, studies investigating the benefit of alternative OSA therapies other than CPAP in BP and other cardiovascular health outcomes are necessary. Several studies have suggested that the beneficial effect of mandibular advancement device may not be inferior [50]. The effect of positional therapy, a highly underappreciated therapy, on BP requires further scrutiny [104]. The investigation of the effect of non-CPAP therapy on BP is important given the poor CPAP compliance and the emergence of novel therapies [105–107].

## Conclusion

OSA is an important contributor to the pathophysiology of hypertension. The role of OSA has been most well recognized in nocturnal as well as resistant hypertension. The fact that OSA is so common in otherwise healthy individuals raises a question about to what extent OSA needs to be treated. There is some evidence that those who exhibit symptoms related to OSA may be most vulnerable to the risk of OSA and may benefit the most from OSA therapy. Patients with OSA with hypertension are at risk of cardiac remodeling, through which secondary cardiovascular consequence (e.g., heart failure, atrial fibrillation) many ensue. The relationship between OSA and hypertension is substantially affected by social determinants of health. Future investigations should focus on identifying the social determinants that underly the OSA-hypertension relationship. Advance in BP monitoring technology will likely bring a new level of understanding of the relationship between OSA and BP.

## Data Availability

Not applicable.

## Abbreviations

APBM: Ambulatory BP monitoring
BMI: Body mass index
BP: Blood pressure
BPV: Blood pressure variability
CPAP: Continuous positive airway pressure
EF: Ejection fraction
GLS: Global longitudinal strain
LV: Left ventricle
OSA: Obstructive sleep apnea
RDI: Respiratory Disturbance Index
RH: Resistant hypertension (RH)
RFH: Refractory hypertension (RFH)
RV: Right ventricle
SHHS: Sleep Heart Health Study
VSC: Vitoria Sleep Cohort
WSCS: Wisconsin Sleep Cohort study
